# Stress hyperglycemia, Diabetes mellitus and COVID-19 infection: The impact on newly diagnosed type 1 diabetes

**DOI:** 10.3389/fcdhc.2022.818945

**Published:** 2022-08-29

**Authors:** Ioanna Farakla, Theano Lagousi, Michael Miligkos, Nicolas C. Nicolaides, Ioannis-Anargyros Vasilakis, Maria Mpinou, Maria Dolianiti, Elina Katechaki, Anilia Taliou, Vasiliki Spoulou, Christina Kanaka-Gantenbein

**Affiliations:** ^1^ Diabetes Center, Division of Endocrinology, Metabolism and Diabetes, First Department of Pediatrics, National and Kapodistrian University of Athens Medical School, “Aghia Sophia” Children’s Hospital, Athens, Greece; ^2^ Immunobiology Research Laboratory and Infectious Diseases Department “MAKKA”, First Department of Pediatrics, National and Kapodistrian University of Athens Medical School, “Aghia Sophia” Children’s Hospital, Athens, Greece

**Keywords:** COVID-19, Type 1 diabetes, diabetic ketoacidosis, hyperglycemia, stress hyperglycemia

## Abstract

Several recent studies have documented an increased incidence of newly diagnosed type 1 Diabetes (T1D) cases in children and adolescents during the COVID-19 pandemic and a more severe presentation at diabetes onset. In this descriptive study, we present the experience of the Diabetes Centre of the Division of Endocrinology, Diabetes, and Metabolism of the First Department of Pediatrics of the National and Kapodistrian University of Athens Medical School at “Aghia Sophia” Children’s Hospital in Athens, Greece, concerning new cases of T1D diagnosis during the COVID-19 pandemic (March 2020- December 2021). Patients who had already been diagnosed with T1D and needed hospitalization due to poor control during the pandemic have been excluded from this study. Eighty- three children and adolescents with a mean age of 8,5 ± 4.02 years were admitted to the hospital due to newly diagnosed T1D during this 22 months’ period in comparison to 34 new cases in the previous year. All patients admitted during the pandemic with a new diagnosis of T1D, presented in their majority with DKA (Ph: 7.2) representing an increase of new severe cases in comparison to previous years (Ph 7.2 versus 7.3, p value: 0.021, in the previous year), [p-value: 0.027]. 49 cases presented with DKA, of which 24 were characterized moderate and 14 severe DKA (28.9% and 16,9%, respectively), while 5 patients newly diagnosed, needed to be admitted to the ICU to recover from severe acidosis. Whether a previous COVID- 19 infection could have been the triggering factor is not supported by the SARS-Cov2 specific antibodies analysis in our cohort of patients. As far as HbA1c is concerned there was no statistically significant difference between the pre COVID-19 year and the years of the pandemic (11.6% versus 11.9%, p- value: 0.461). Triglycerides values were significantly higher in patients with new onset T1D during COVID-19 years compared to those before the pandemic (p value= 0.032). Additionally, there is a statistically significant correlation between Ph and Triglycerides for the whole period 2020-2021 (p-value<0.001), while this correlation is not significant for the year 2019. More large- scale studies are required to confirm these observations.

## Introduction

The Corona Virus Disease-19 (COVID-19) has been first described as a severe form of pneumonia reported in Wuhan, China in the late 2019 (1). The COVID-19 has been subsequently spread worldwide, so that on March 11th, 2020, the World Health Organization has declared it as a pandemic (2). Since the initial description of the disease, caused by the SARS-CoV2 virus, more that 6 million people worldwide lost their lives (3), and the disease is continuously spreading worldwide with a frightening velocity, while new mutations account for the continuously evolving clinical outcome (4).

Several underlying medical conditions, such as chronic pulmonary disease, arterial hypertension, cardiovascular events but also obesity and Type 2 Diabetes (T2D) pose an increased risk for severe COVID-19 outcome. The severe course of the COVID-19 infection in case of T2D is mainly attributed to the inflammatory state that characterizes obese patients with T2D leading to aggravation of insulin resistance, hyperglycemia with resulting glucotoxicity, increased oxidative stress and endothelial dysfunction, all contributing to increased morbidity (5). On the other hand, it was observed that young patients with T1D were not represented among the patients affected by COVID-19 and, therefore, many articles appeared, especially during the first waves of the pandemic, suggesting that patients with T1D may even be spared from the SARS-CoV2 infection (6).

Moreover, most countries have implemented a range of public health care interventions and government regulations to mitigate the transmission of SARS-CoV-2. This situation significantly reduced pediatric emergency department (ED) access, most likely due to the fear of infection of their children, while awaiting to get examined (7). As a result, Based on international reports from pediatric hospitals, it has been observed that newly diagnosed cases of T1D came to medical attention or seek medical care at a more advanced stage with severe dehydration and ketoacidosis and it was concluded that during the COVID-19 pandemic patients were reluctant to attend hospitals due to the fear of contamination with SARS-CoV2. Furthermore, there is a universal concern that the new coronavirus SARS-CoV2 may induce the autoimmune destruction of insulin-producing pancreatic beta cells through binding to the ACE-2 receptor expressed on the surface of the pancreatic beta cells, representing thus an environmental triggering factor for T1D initiation (8–11).

Diabetic ketoacidosis (DKA) is an avoidable complication of T1D if signs and symptoms of diabetes mellitus are recognized early, and the goal of the medical community worldwide is to diagnose T1D at an early stage before the exhaustion of beta cell reserve and the resulting occurrence of severe ketoacidosis (12). DKA not only places increased burden on the healthcare system and, in some cases, may even require intensive care support in a tertiary care setting, but, more importantly, is associated with advanced exhaustion of the beta cell secretory capacity, associated with an earlier occurrence of long-term diabetes complications (13). Moreover, during the pandemic period, it was important to minimize avoidable admissions to intensive care units, when the public health system worldwide was meant to spare medical staff and ICU coverage for the patients affected by severe COVID-19. Studies during the COVID-19 pandemic have reported an increase in pediatric cases of T1D presenting with DKA in many countries such as Italy, UK, USA, Canada, Southern Turkey (9–11, 14–23). A study in an Australian pediatric tertiary center reported a significant increase in presentation of severe DKA in a paediatric population with newly diagnosed T1D during the Covid -19 pandemic (11).

The aim of the current study was therefore to present the experience of the Diabetes Centre of the Division of Endocrinology, Diabetes, and Metabolism of the First Department of Pediatrics of the National and Kapodistrian University of Athens Medical School at “Aghia Sophia” Children’s Hospital in Athens, Greece, regarding both the incidence as well as the severity of new cases of T1D during the first two years of the COVID-19 pandemic, namely from March 2020 to December 2021, in comparison to the previous year 2019 and, furthermore, to compare these data with the existing literature data from other countries during the pandemic.

## Patients and methods

### Patients cohort

All children and adolescents aged <18 years with the initial diagnosis of T1D hospitalized at the “Aghia Sophia” Children’s Hospital (Athens, Greece) between March 2020 and December 2021 were included in the study. The number and data concerning DKA presentation of these patients were compared to the respective cases of the 12 months’ period preceding the pandemic.

### Ethical considerations

The study was approved by the “Aghia Sophia” Children’s Hospital Ethics Committee for Human Research. Written informed consent was obtained from the parents of the patients before their participation in the study.

### Clinical and laboratory parameters

Demographic and clinical data (age, date at diagnosis, personal and family history for severe diseases, family history of T1D), as well as auxological data, including height, weight, body mass index (BMI) and pubertal stage of the participants were collected. Endocrinological and biochemical data including HbA1c, initial venous blood gas analysis (pH, bicarbonate), serum glucose, C-peptide, and type 1 diabetes-associated antibodies [anti-glutamic acid decarboxylase antibodies, islet antigen 2, islet cell and insulin antibodies] were also determined. Based on the International guidelines of the International Society for pediatric and Adolescent diabetology (ISPAD) on DKA (24), DKA was categorized into three groups according to the severity: mild (pH: 7,2-7,3 and/or bicarbonate: 10-15 mmol/L), moderate: (pH: 7,1-7,2 and/or bicarbonate: 5-10 mmol/L) and severe (pH: <7,1 and/or bicarbonate: <5 mmol/L). The autoimmune etiology was confirmed by the presence of at least two or more autoantibodies for T1D.

### Serum detection of antibodies against the SARS-CoV2

To investigate whether the newly diagnosed patients with T1D have been previously infected with the SARS-CoV2 virus, antibodies against the SARS-CoV2 were assessed in all patients through ELISA by the use of an “in-house” ELISA, using the combination of the Receptor Binding Domain (RBD) and S2 domain within S protein and the whole N protein as capture antigens. This “in-house” ELISA has been previously developed in our Laboratory, with comparable, and even higher, sensitivity and specificity with other commercially available ELISA kits (92% and 97% respectively) (26).

Briefly, 96-well plates (Nunc Maxisorp, Rochester, NY, USA) were coated with RBD 2.5 µg/mL, S2 1 µg/mL and N 1.5 µg/mL suspended in Phosphate Buffered Saline (PBS). After blocking with PBS containing 2% Bovine Serum Albumin (BSA) at 37°C for 30 minutes, diluted serum samples (n=65), in a dilution 1/100 in 2% BSA PBS were added and incubated for 1 hour at 37°C. Each serum sample was also evaluated against BSA (0.01% PBS) to eliminate non-specific binding. Alkaline phosphatase conjugated goat anti-human IgG (Jackson ImmunoResearch Laboratories, 1/3000) antibody diluted in PBS/BSA was used to reveal specific human antibodies (IgG). Antibody-binding was assessed with the substrate 4-nitrophenyl-phosphate-disodium salt hexahydrate (Sigma Chemicals) at 405 nm (Chromate reader, Awareness Technology). R3022, a human SARS-CoV antibody previously determined to cross-react with SARS-CoV-2 was used as a positive control. The cut-off value has been previously determined as the mean plus 2 standard deviations (SD) of a pool of pre-COVID-19 controls (26). Age-matched healthy children who were regularly followed-up at the Pediatric Department of our Hospital were used as controls (n=120). Data was analyzed in Prism (GraphPad).

### Statistical analysis

Qualitative variables are presented as absolute and relative (%) frequencies. Quantitative variables are presented using measures of location (i.e. mean, median) and measures of dispersion (i.e. SD, min, max). To compare qualitative variables between the 2 groups, chi-square test (exact) was implemented. For quantitative variables, we used Mann-Whitney test for between groups comparisons.

A two-tailed p-value<0.05 was considered statistically significant. For the statistical analysis we used IBM SPSS v. 26 (IBM Corp. Released 2019. IBM SPSS Statistics for Windows, Version 26.0. Armonk, NY: IBM Corp.).

## Results

### Clinical and laboratory characteristics

A total of 83 children (42 boys, 52,1%), with a mean age of 8.5 years (± 4.02 years) had been hospitalized at the “Aghia Sophia” Children’s Hospital between March 2020 and December 2021, i.e., during the 22 months covering the 3 waves of the pandemic period, while during the preceding pre-pandemic year (2019) a total of 35 children with the new diagnosis of T1D had been admitted to the hospital (25 boys, 71.4%). Of the 83 patients newly diagnosed with T1D during the 22 months’ period of the pandemic from March 2020 to December 2021, DKA was present in 53 participants (63,9%), that was not statistically different to the DKA percentage of the pre- covid period (48.6%), [p value: 0.151]. Among the patients who presented with DKA at T1D onset, 11 patients presented with mild DKA (13,3%), 24 with moderate DKA (28,9%) and 14 patients with severe DKA (16,9%) (**Table 1**) concerning four patients who have been initially diagnosed with diabetic ketoacidosis in other health centers and were subsequently transferred to our hospital for further treatment and diabetes education, unfortunately no data on their initial blood gas were available in order to classify them in a group of mild, moderate or severe DKA. It is worth mentioning that 5 of the participants necessitated hospitalization in the Intensive Care Unit (ICU) due to their severe ketoacidosis and poor general condition.

**Table 1:** Demographic and clinical characteristics of children and adolescents admitted with DKA: quantitative variables (Ν = 118).

Variables	Group	Levels	Ν	%	p-value^*^
Gender	2019	Male	25	71.4	**0.043**
		Female	10	28.6	
	2020-21	Male	42	50.6	
		Female	41	49.4	
Season	2019	1	8	22.9	0.072
		2	5	14.3	
		3	7	20.0	
		4	11	31.4	
		Missing values	4	11.4	
	2020-21	1	20	24.1	
		2	24	28.9	
		3	26	31.3	
		4	12	14.5	
		Missing values	1	1.2	
DKA	2019	Yes	17	48.6	0.151
		No	17	48.6	
		Missing values	1	2.9	
	2020-21	Yes	53	63.9	
		No	29	34.9	
		Missing values	1	1.2	
DKA severity	2019	0	17	48.6	0.219
		1	6	17.1	
		2	7	20	
		3	2	5.7	
		Missing values	3	8.6	
	2020-21	0	29	34.9	
		1	11	13.3	
		2	24	28.9	
		3	14	16.9	
		Missing values	5	6	
antiGAD < 5	2019	Positive	19	54.3	1.000
		Negative	9	25.7	
		Missing values	7	20	
	2020-21	Positive	53	63.9	
		Negative	23	27.7	
		Missing values	7	8.4	
IAA	2019	Positive	20	57.1	0.181
		Negative	8	22.9	
		Missing values	7	20	
	2020-21	Positive	44	53	
		Negative	35	42.2	
		Missing values	4	4.8	
ICA	2019	Positive	13	37.1	0.501
		Negative	16	45.7	
		Missing values	6	17.1	
	2020-21	Positive	25	30.1	
		Negative	43	51.8	
		Missing values	15	18.1	
ZnT8	2019	Positive	10	28.6	0.281
		Negative	9	25.7	
		Missing values	16	45.7	
	2020-21	Positive	46	55.4	
		Negative	22	26.5	
		Missing values	15	18.1	

^*^: Chi-square test was used for the comparisons between the 2 groups.

### Seasonality of new diagnosis of T1D before and during the pandemic

**Figure 1:**
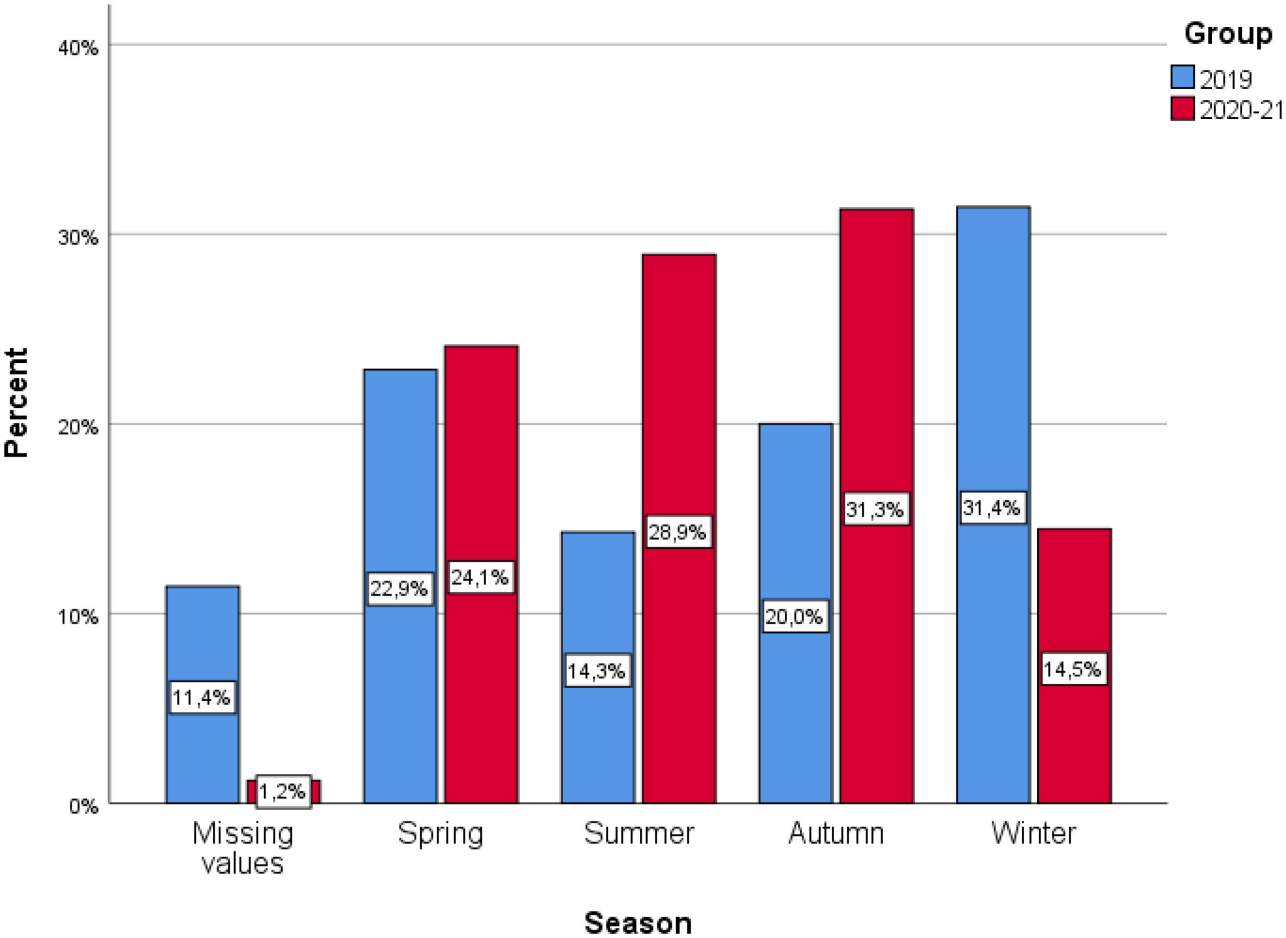
Boxplot of Ph for the 2 groups of interest. P-value: 0,021.

**Figure 2:**
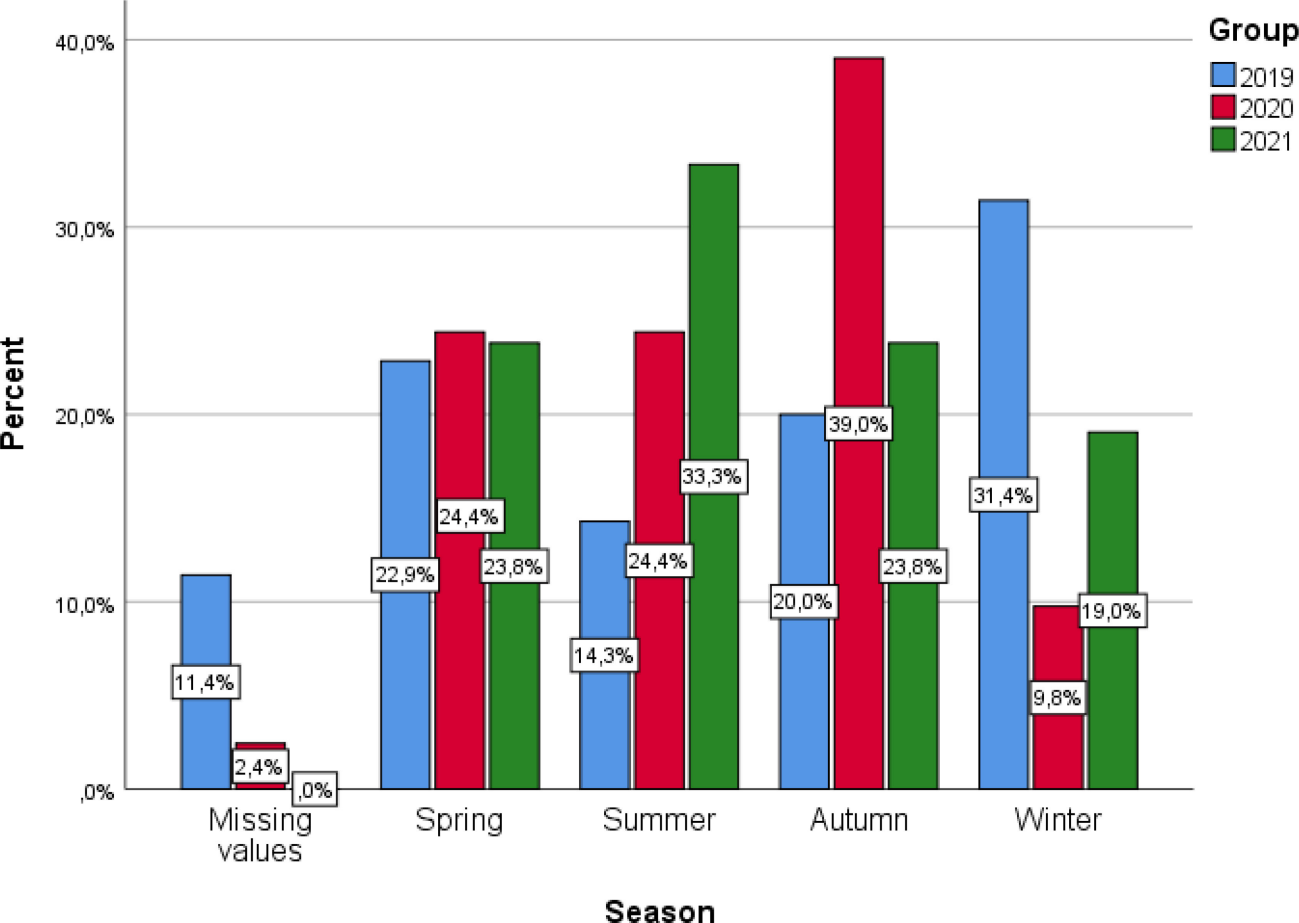
Boxplot of Tg (triglycerides) for the 2 groups of interest. P-value: 0,032. Values with a circle denote outliers, while values with an asterisk denote extreme outliers.

We studied the seasonality of new diagnosis of T1D one year before and for 22 months’ period during the pandemic as a whole and separately. An increase of new cases of T1D during summer and autumn was observed, as far as the pandemic years are concerned (28.9% & 31.3% respectively) versus pre-COVID19 year (14.3% & 20%). In contrast, based on the data from the pre-COVID19 year, most of the cases with new onset T1D were documented during winter for the last year prior to the pandemic (31.4% vs 14.5%) (**Figure 1**). Furthermore, we observed differences in seasonality within the two years of the pandemic. In the first year, the increased frequency was noted during autumn (39%), while in the second year most cases presented during summer (33.3%) (**Figure 2**).

### Baseline laboratory characteristics before and during the pandemic

**Figure 3:**
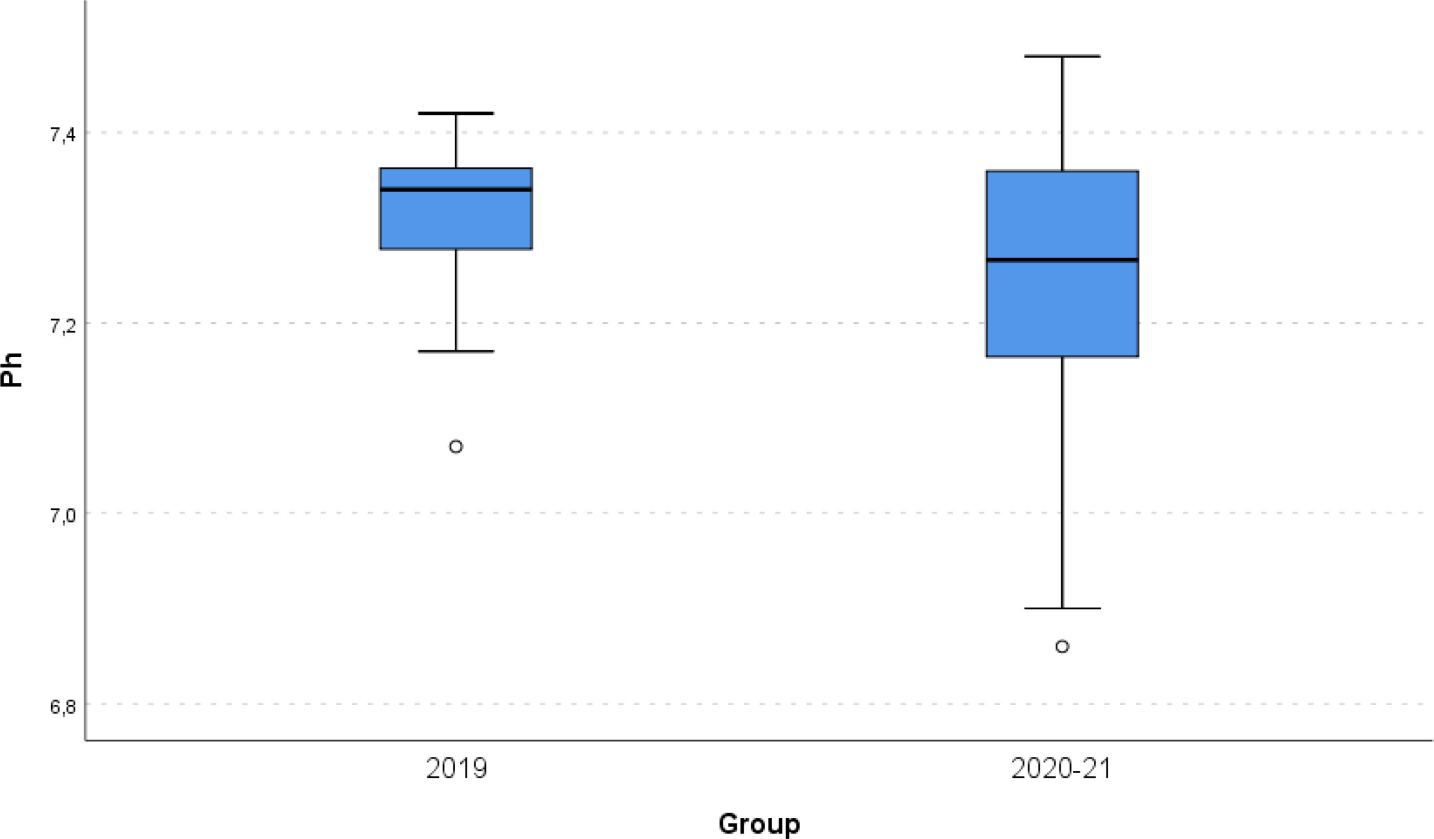
Barplot of season for the 2 groups of interest.

**Figure 4:**
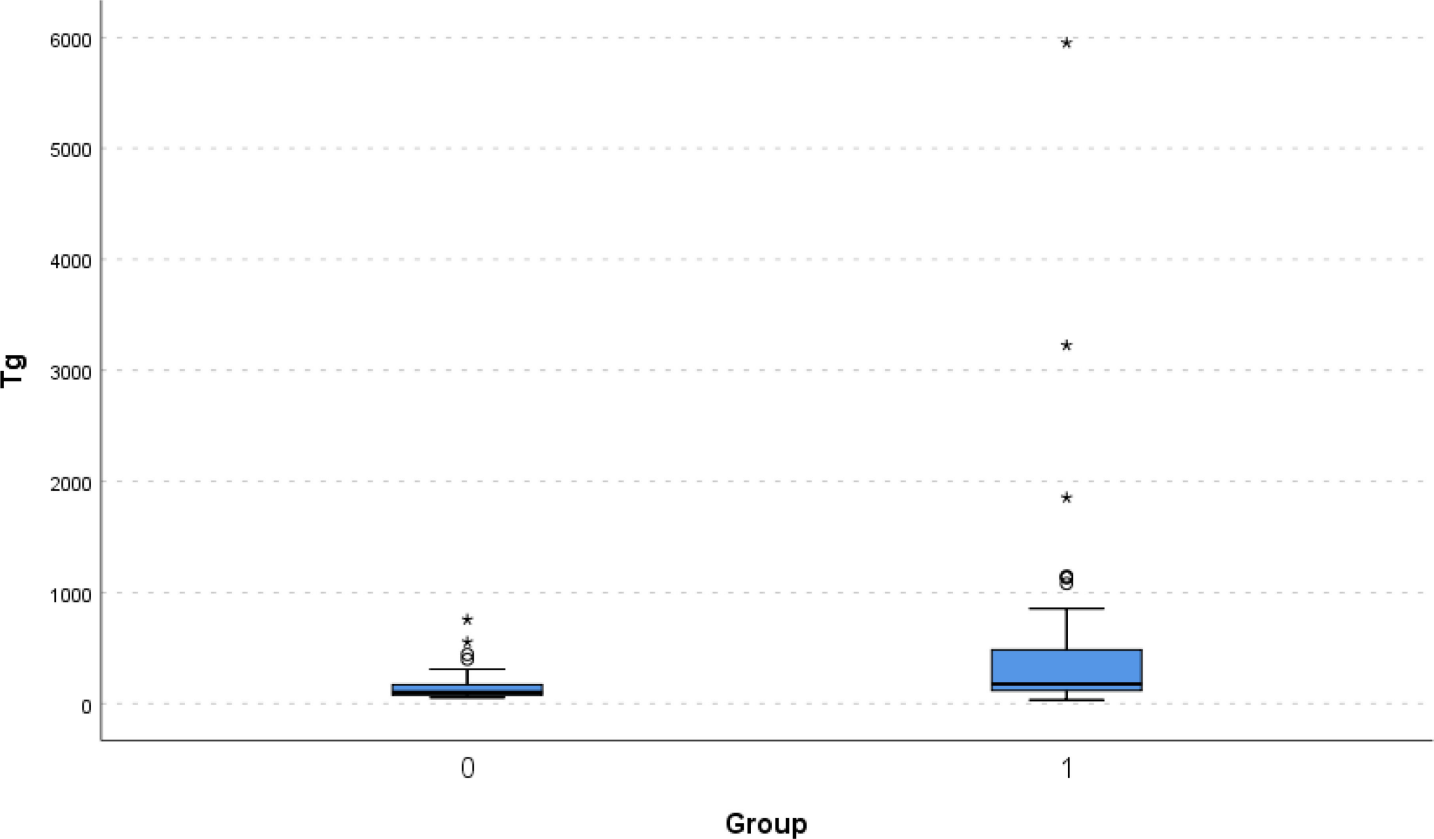
Barplot of season for the 3 groups of interest.

No differences were found regarding total cholesterol (CHOL), HDL and LDL (**Table 2**). There were statistically significant differences in Ph and Triglycerides (Tg) between the two groups. In specific, Ph was lower in the pandemic group (p-value: 0.021), while Tg were higher in the pandemic group (p- value: 0.032) (**Table 2**). There is a statistically significant correlation between Ph and Tg for the Covid-19 period (p value < 0.001) while this is not significant for year 2019 (p value: 0.387) (**Table 3** and **Figures 3**, **4**). As far as HbA1c is concerned there was no statistically significant difference between the pre COVID-19 year and the years of the pandemic (11.6% versus 11.9%, p- value: 0.461). (**Table 2**)

**Table 2:** Demographic and clinical characteristics of the sample: quantitative variables (Ν = 118).

Variable	Group	N	Mean	Median	SD	Min	Max	p-value^*^
Age	2019	34	9.1	9.0	4.09	2.0	16.0	0.424
	2020-21	83	8.5	8.0	4.02	1.0	16.0	
	Total	117	8.7	9.0	4.03	1.0	16.0	
Ph	2019	31	7.3	7.3	0.09	7.1	7.4	**0.021**
	2020-21	72	7.2	7.3	0.15	6.9	7.5	
	Total	103	7.3	7.3	0.13	6.9	7.5	
CHOL	2019	24	185.0	170.5	68.12	113.0	427.0	0.444
	2020-21	60	197.9	188.5	72.25	79.0	441.0	
	Total	84	194.2	179.0	70.92	79.0	441.0	
TG	2019	23	187.3	100.0	183.05	55.0	756.0	**0.032**
	2020-21	58	483.0	176.0	896.97	30.0	5950.0	
	Total	81	399.0	155.0	774.90	30.0	5950.0	
LDL	2019	22	101.0	101.5	37.43	41.0	219.0	0.247
	2020-21	51	92.1	86.0	48.65	21.0	217.0	
	Total	73	94.8	91.0	45.49	21.0	219.0	
HDL	2019	24	47.9	41.0	23.09	29.0	129.0	0.857
	2020-21	52	44.6	42.5	18.46	18.0	111.0	
	Total	76	45.6	41.0	19.94	18.0	129.0	
HBA1C	2019	32	11.6	12.0	2.19	6.0	18.0	0.461
	2020-21	75	11.9	11.8	2.45	5.5	16.3	
	Total	107	11.8	11.9	2.36	5.5	18.0	

^*^ Mann-Whitney test was used for the comparisons between the 2 groups.

**Table 3:** Spearman correlation coefficient results between blood Ph and Triglycerides for the whole period and separately for each period.

Period	rho	p-value
2019	-0.199	0.387
2020-21	-0.569	**<0.001**
2019-21	-0.554	**<0.001**

There is a statistically significant correlation between Ph and Triglycerides for the whole period (p<0.001) and for the period 2020-21 (p<0.001). This correlation is not significant for year 2019.

**Figure 5:**
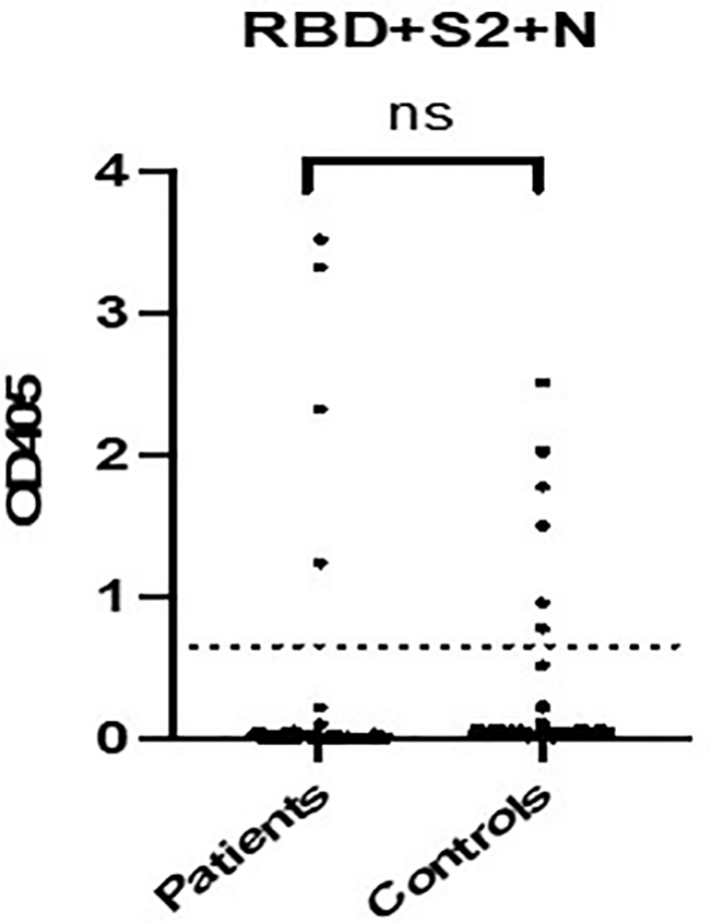
Prevalence of antibodies against the combination of the RBD and S2 domain within S protein and the whole N protein in sera from 65 patients and 120 controls, using the “in-house” ELISA. The cut-off value had been previously determined using the mean optical density plus 2 standard deviations (SDs) of pre-pandemic controls, represented by the dotted line. Black symbols represent the antibody-binding as Optical Density (OD) value of each serum at 405 nm. The rate of positivity did not differ significantly between the two groups (p >0.05). ns: Not Statistically Significant.

During the pandemic, all patients tested for Covid- 19 with rapid antigen test at admission and antibodies against SARS-CoV-2 were assessed. If they had symptoms suggesting Covid -19 disease (like fever, abdominal pain, vomiting, etc.) they underwent PCR for SARS- CoV-2. Rapid tests were negative for all patients. Immunoactivity against SARS-COV2 in patients with newly diagnosed T1D and healthy children was evaluated using an “in house” ELISA where the combination of three different antigens (RBN, S2 and N) in their soluble form was used as capture antigens (26). Three patients out of the 83 that were evaluated recognized Covid -19 antigens and were classified as positive, implying that they have successfully recovered from COVID-19 disease 10 days to 3 weeks prior to the admission for T1D. The SARS – COV2 antibody test was positive for six controls (out of 120 assessed) as well. Three of them were confirmed to have positive PCR, prior to admission, while the rest of them denied experiencing COVID-19 symptoms, implying that this positivity was most likely associated with the previous asymptomatic infection. The rate of positivity did not significantly differ between the two groups (p>0.05). Notably, 3 patients were considered negative for COVID-19 based on ELISA results, although they had a positive PCR-test confirming a recent COVID-19 disease upon admission. This discrepancy between the positive PCR testing and the negative SARS-CoV2 antibodies titer may be explained as most COVID-19 patients elicit specific antibodies against SARS- CoV 2 14 days post disease onset, although several discrepancies have been reported (27) (**Figure 5**).

## Discussion

In the current study, we present the experience of the Diabetes Centre of the Division of Endocrinology, Diabetes, and Metabolism of the First Department of Pediatrics of the National and Kapodistrian University of Athens Medical School at “Aghia Sophia” Children’s Hospital in Athens, Greece, regarding number and severity of newly diagnosed children and adolescents with T1D during the first 22 months’ period of the COVID-19 pandemic. From March 2020 until December 2021, 83 children and adolescents with a mean age of 8.5 years (± 4.2 years) were admitted to the hospital due to newly diagnosed T1D. The absolute number of newly diagnosed cases of T1D was higher compared to that of the previous 12 months (83 new cases in 2020-2021, in more detail 42 the first year and 41 the second-year, vs 35 cases in 2019). The number of patients with more severe DKA at presentation was not statistically significantly increased (16.9% presenting with DKA in 2020-2021 vs. 5.7% presenting with DKA during the previous non-pandemic year, p- value: 0.219) although the mean value of blood pH during the 2020-2021 22 months' pandemic period (pH: 7.2) was statistically lower that the mean blood pH value (pH: 7.3) of the pre covid- 19 year.

Our findings concerning the severity of DKA at presentation of new cases of T1D do not agree with those of similar studies carried out in many European and non-European countries during the COVID-19 pandemic reporting an increase of pediatric cases of T1D presenting with DKA like Italy, Germany, UK, USA, Southern Turkey, and Australia (9, 10, 14–24, 28–52). This apparent discrepancy between our observation and the literature data concerning severity DKA at presentation could probably be attributed to the smaller sample size of our cohort not allowing to unravel subtle differences. Since the observation of a greater severity of DKA diabetes onset as well as presumed overall increase of new T1D cases following the pandemic year is still an evolving phenomenon, only large scale multi-center studies could better clarify this issue in the future. Nevertheless, we have also observed that Ph on admission was statistically significantly lower during the pandemic years. (p value = 0.021). (**Figure 3**; **Table 2**) The majority of the 83 patients hospitalized in our hospital with a new diagnosis of T1D, mainly presented with DKA (63.9%). Most DKA cases had moderate or severe DKA (28,9% and 16,9%, respectively), while 5 patients needed to be admitted to the ICU to recover from severe acidosis. No differences were found regarding auto- antibodies for T1D since all newly diagnosed patients with T1D demonstrated positivity for at least two T1D-specific autoantibodies. (**Table 1**) Whether a previous COVID- 19 infection could have been the triggering factor is not supported by the SARS-CoV2 specific antibodies analysis in our cohort of patients. The SARS-CoV2 antibody test was positive in 3 patients with T1D and 6 controls, therefore demonstrating that the positivity rate did not significantly differ between the two groups. Thus, our data did not support a clear association between previous SARS-CoV2 infection and T1D onset, similarly to previous reports (53). (**Figure 5**) Not only infectious agents but also psychosocial stressors are known to play a fundamental role in the pathophysiology of T1D initiation, since several physical catastrophic events have been reported to be followed by an increased number of newly diagnosed cases of T1D (54, 55). However, in the current study no questionnaires investigating the psychological impact of the pandemic per se but also of the lockdown have been used, and thus no conclusions on that topic can be drawn. We speculate that public health measures implemented during the pandemic may have provoked stress, anxiety and fear to the children and adolescents and resulted to the emergence of autoimmune-mediated diabetes initiation, however, no data from the current study are available to assess such a possible association.

In our study, we have observed a more aggressive progress of T1D during the pandemic, since 5 of the participants needed to be admitted to the ICU department in contrast with only one patient the year before the pandemic. Two studies, so far, have documented that DKA presentation was more severe during the pandemic, compared to the pre-COVID-19 period (9, 52). This observation might be attributed to the delay of patients seeking medical care and attending hospitals due to the fear of contamination with SARS-CoV2 and/or the possible more severe autoimmunity.

Previous studies have shown an association between lipid profile impairment and T1D onset (56). In our study, triglycerides values were significantly higher in patients with new onset T1D during COVID-19 years compared to those before the pandemic (p value= 0.032). (**Figure 4**; **Table 2**) Additionally, there is a statistically significant correlation between Ph and Tg for the whole period 2020-2021 (p-value<0.001), while this correlation is not significant for the year 2019. Total, LDL and HDL cholesterol were similar between the pandemic period and the pre- pandemic period. The same findings of a significant negative correlation between blood pH and triglycerides levels during the pandemic years in the absence of such a correlation in the preceding year have already been reported by Mastromauro C., et al. (57) pointing towards an additional role of lifestyle factors during the pandemic as an aggravating factor for the triglycerides’ elevation. Based on both our own data as well as the data reported from the Italian experience, it might, therefore, be suggested that there is not only an association between lipid profile impairment and severity of DKA in newly diagnosed T1D, but, furthermore, that also lifestyle changes during the COVID- 19 pandemic might have affected the lipid profile of newly diagnosed T1D cases.

Furthermore, according to these accumulated data, in our cohort an increased number of newly diagnosed cases of T1D was observed during the summer and autumn months. Moreover, we observed differences in seasonality during the pandemic period in comparison to the pre-pandemic year. (**Figures 1**, **2**) In the first year, the increased frequency was observed during autumn (39%), after the first wave, while in the second year most cases presented during summer (33.3%), after the second wave. So, after the first wave there was a time lag of around 6 months between the initiation of the pandemic and the newly diagnosed T1D cases, resulting at a peak of newly diagnosed T1D cases during autumn, while, after the second wave of the pandemic, there was another peak in the incidence of new T1D cases after a lag time of around 6-8 months. It is well known that a clear elapse time between the effect of a triggering factor for the initiation of the beta cell destruction of at least 6 months up to two years is occurring until the overt diabetes onset. Therefore, it is quite plausible that the first wave of the pandemic resulted in the first peak of T1D onset after a time lag period of around 6 months, while the second peak in the occurrence of new cases of T1D may both be attributed to previous COVID-19 infection that occurred during the previous 6-15 months before the diabetes onset. Although such an elapse time fits well to the time reported between the triggering factor and diabetes initiation, however, the lack of documentation of a previous infection with the SARS-CoV2 virus, as suggested by the non-significant presence of SARS-CoV2 antibodies among the patients who presented with new T1D, renders the previous SARS-CoV2 infection less probable to initiate the autoimmune destruction of the beta cells. Although several studies have attempted to demonstrate a pattern of seasonality, collected data are still controversial. Nevertheless, it has been reported that the incidence rate of new diagnosis of T1D reaches a peak during the winter months as was also the case in our new T1D patients in the year preceding the pandemic (58). According to the previous and current data from our institution the seasonal distribution of diagnosis of T1D during the pandemic has changed and perhaps depends on the severity of the pandemic. In a recent study from Greece, Kostopoulou et al. investigated the impact of COVID-19 on new-onset of T1D in children and adolescents hospitalized in the two children’s hospitals in Patras, Greece, during the pandemic. They found a gradual increase of the incidence of new diagnosis of T1D from spring to winter, compared to the pre-COVID-19 year, where spring and autumn months displayed the lower rate of newly diagnosed cases of T1D. We may further speculate that the increased number of T1D diagnosis during autumn may have occurred since the parents of the patients were reluctant to attend hospitals due to fear of contamination with SARS-CoV2 even for routine blood exams and attended the hospitals at a more advanced stage of diabetes. Indeed, several studies have reported a reduction in the number of visits at the Emergency Department both for children and adults during the first wave of the pandemic (25, 52, 59, 60).

Our study has several limitations. There is a limited number of participants from a single pediatric center in Athens, Greece; therefore, no safe conclusions can be drawn.

Furthermore, the cellular and molecular involvement of SARS-CoV2 infection in the pathogenesis of T1D could not be confirmed by the implementation of an “in house” ELISA with comparable sensitivity with commercially available SARS- COV2 diagnostic ELISA kits (50) that has been used to assess seroprevalence of SARS- COV2 in our patients. **[** **Figure 5** **]** Therefore, further, larger, and multicenter studies are needed to draw safe conclusions. Nevertheless, our study showed a different pattern of seasonality of T1D cases and a rather severe presentation of new cases of T1D. The elapse time between the COVID-19 pandemic waves and the higher incidence of new T1D cases ranged from 6 to 9 months, a period already reported between the effect of a triggering factor to the outbreak of overt diabetes.

In conclusion, we could not confirm a statistically significant higher incidence of newly diagnosed T1D cases during the pandemic, although a trend was obvious, but a seasonality pattern that is reminiscent of a lag period of 6-9 months between the separate COVID-19 waves and the respective peaks in the incidence of new T1D cases has been observed. Although we could not confirm an etiological link between previous SARS-CoV2 infection and resulting diabetes autoimmunity, as witnessed by the lack of SARS-CoV2-specific seropositivity, it is however plausible that the psychological burden of the lockdown and social isolation imposed to children during these individual waves of the pandemic may have triggered beta cell auto-immunity that resulted in new T1D onset.

Since the possible association between the Covid -19 pandemic and T1D cases is still an envolving phenomenon, only prospective, multicenter, large-scale studies could clarify this issue in the future.

## Data availability statement

The raw data supporting the conclusions of this article will be made available by the authors, without undue reservation.

## Ethics statement

The studies involving human participants were reviewed and approved by The Ethics committee of the Aghia Sophia Children’s Hospital, Athens, Greece. Written informed consent to participate in this study was provided by the participants’ legal guardian/next of kin.

## Author contributions

CKG and IF conceived and designed the study. IF coordinated the participants’ recruitment. IF, NN, I-AV, MMp, MD, EK and AT contributed to the collection of clinical characteristics, as well as endocrinological and biochemical parameters. TL performed the ELISA experiments under the supervision of VS. MMi performed the statistical analysis. NN, IF, TL and MMi wrote the original draft of the manuscript. CKG edited the final version of the manuscript. All authors approved the submitted version.

## Acknowledgments

Special thanks to the families of the patients and the patients *per se*.

## Conflict of interest

The authors declare that the research was conducted in the absence of any commercial or financial relationships that could be construed as a potential conflict of interest.

## Publisher’s note

All claims expressed in this article are solely those of the authors and do not necessarily represent those of their affiliated organizations, or those of the publisher, the editors and the reviewers. Any product that may be evaluated in this article, or claim that may be made by its manufacturer, is not guaranteed or endorsed by the publisher.
